# The effect of short-term high versus normal protein intake on whole-body protein synthesis and balance in children following cardiac surgery: a randomized double-blind controlled clinical trial

**DOI:** 10.1186/s12937-015-0061-9

**Published:** 2015-07-28

**Authors:** Vincent G. Geukers, Monique E. Dijsselhof, Nicolaas J. G. Jansen, Johannes M. P. J. Breur, Dewi van Harskamp, Henk Schierbeek, Johannes B. van Goudoever, Albert P. Bos, Hans P. Sauerwein

**Affiliations:** 1 Pediatric Intensive Care Department H8-190, Emma Children’s Hospital (EKZ), Academic Medical Center (AMC), Meibergdreef 9, 1105 AZ Amsterdam, The Netherlands; 2 Department of Clinical Nutrition, AMC, Amsterdam, The Netherlands; 3 Pediatric Intensive Care Department, Wilhelmina Children’s Hospital (WKZ), University Medical Center Utrecht (UMCUtrecht), Lundlaan 6, 3584 EA Utrecht, The Netherlands; 4 Department of Pediatric Cardiology, WKZ, UMCUtrecht, Utrecht, The Netherlands; 5 Department of Pediatrics, EKZ, AMC, Amsterdam, The Netherlands; 6 Department of Pediatrics, VU University Medical Center, Boelelaan 1117, 1081 HV Amsterdam, The Netherlands; 7 Department of Endocrinology and Metabolism (emeritus), AMC, Amsterdam, The Netherlands

**Keywords:** Proteins, Isotopes, Child, Congenital heart defect, Intensive care

## Abstract

**Background:**

Infants undergoing cardiac surgery are at risk of a negative protein balance, due to increased proteolysis in response to surgery and the cardiopulmonary bypass circuit, and limited intake. The aim of the study was to quantify the effect on protein kinetics of a short-term high-protein (HP) diet in infants following cardiac surgery.

**Methods:**

In a prospective, double-blinded, randomized trial we compared the effects of a HP (5 g · kg^−1^ · d^−1^) versus normal protein (NP, 2 g · kg^−1^ · d^−1^) enteral diet on protein kinetics in children <24 months, on day 2 following surgical repair of congenital heart disease. Valine kinetics and fractional albumin synthesis rate (FSR_alb_) were measured with mass spectrometry using [1-^13^C]valine infusion. The Mann–Whitney *U* test was used to investigate differences between group medians. Additionally, the Hodges-Lehmann procedure was used to create a confidence interval with a point estimate of median differences between groups.

**Results:**

Twenty-eight children (median age 9 months, median weight 7 kg) participated in the study, of whom in only 20 subjects isotopic data could be used for final calculations. Due to underpowering of our study, we could not draw conclusions on the primary outcome parameters. We observed valine synthesis rate of 2.73 (range: 0.94 to 3.36) and 2.26 (1.85 to 2.73) μmol · kg^−1^ · min^−1^ in the HP and NP diet, respectively. The net valine balance was 0.54 (−0.73 to 1.75) and 0.24 (−0.20 to 0.63) μmol · kg^−1^ · min^−1^ in the HP and NP group. Between groups, there was no difference in FSR_alb_. We observed increased oxidation and BUN in the HP diet, compared to the NP diet, as a plausible explanation of the metabolic fate of surplus protein.

**Conclusions:**

It is plausible that the surplus protein in the HP group has caused the increase of valine oxidation and ureagenesis, compared to the NP group. Because too few patients had completed the study, we were unable to draw conclusions on the effect of a HP diet on protein synthesis and balance. We present our results as new hypothesis generating data.

**Trial registration:**

Dutch Trial Register NTR2334.

## Background

Critically ill children on a Pediatric Intensive Care Unit (PICU) usually receive less than recommended protein intake, with 50 % of cumulative deficits developing in the first 48 h of admission [1]. As a result, lean body mass (LBM) deteriorates during the admission period, superimposed on the fact that approximately a quarter of patients is already undernourished on admission [1, 2]. Especially infants and young children after cardiac surgery, due to higher metabolic demands and fluid restriction, may be at risk of undernutrition [3]. Poor pre-operative nutritional status with further decrease of LBM during admission increases morbidity, infection rate, and length of stay (LOS) in the ICU [4].

In pediatric surgical patients, increased endogenous proteolysis with negative net whole-body protein balance, is the result of an endocrine and inflammatory response to surgery [5]. Additionally, in cardiac surgical procedures, the use of a cardiopulmonary bypass circuit (CPB) induces complement activation, endotoxin release, leukocyte activation, and the release of many pro-inflammatory mediators, adding to the stress response [6].

The importance of early enteral nutrition (EN) in the PICU population was underlined by a recent multi-center trial including > 5000 critically ill children from 12 North-American PICU’s with LOS of ≥ 96 h, showing a strong and statistically significant association between early EN and lower mortality (odds ratio, 0.51; 95 % confidence interval: 0.34 to 0.76, p = 0.001) [7]. However, data on optimal protein intake in the early phase following pediatric cardiac surgery are lacking. In adult cardiothoracic patients, recommended protein intake is 1.5-2.0 g · kg^−1^ · d^−1^ [8]. In critically ill children, recommended protein intake for children 0–2 years is 2–3 g · kg^−1^ · d^−1^ [9]. Higher protein intake (up to 3 g · kg^−1^ · d^−1^) can further improve protein balance in children with acute illness (e.g. respiratory failure, head injury, sepsis and surgical repair of congenital heart disease) [10]. Based on a previous study in children with cystic fibrosis [11], we hypothesized that whole-body protein synthesis rate and net balance, and albumin synthesis rate, but not endogenous proteolysis, can be improved with protein intake of 5 compared to 2 g · kg^−1^ · d^−1^.

In this prospective, randomized, controlled study in infants after cardiac surgical repair of a low-complex congenital heart defect (CHD), we investigated the short-term (<48 h) effects on whole-body valine kinetics and albumin synthesis rate, of high protein (HP; 5 g · kg^−1^ · d^−1^) dietary intake, compared to normal protein diet (NP; 2 g · kg^−1^ · d^−1^). Also, we studied the effect of the HP diet on the postoperative endocrine (*i.e.*, insulin, cortisol) response.

## Materials and methods

### Subjects

Children with CHD in the pre-operative phase of surgical repair were recruited from the pediatric cardiology departments of the Wilhelmina Children’s Hospital/University Medical Center Utrecht (UMCUtrecht), The Netherlands, Radboud University Medical Center, Nijmegen, The Netherlands and Maximá Medical Center, Veldhoven, The Netherlands. Inclusion criteria were age 3–24 months, and pending low-complex (Aristotle score 1–2 out of 4 [12]), surgical repair of ventricular septal defect (VSD) and/or atrial septal defect (ASD), or partial atrioventricular septal defect (pAVSD) with CPB. Exclusion criteria were: trisomy of chromosome 21, infection (i.e. fever > 38.5 °C for > 4 h with positive blood culture < 48 h), mechanical ventilation or inotropic medication during the period of isotopic infusions, intolerance to enteral tube feeding, and post-operative use of medication with modulating effects on protein metabolism (insulin, steroids). We allowed the use of routine sedation medication (low-dose propofol 10 %) during transport of the mechanically ventilated patient from the operation room (OR) to the PICU until extubation. Additionally, postoperative pain medication (morphine, paracetamol) and diuretics were allowed.

All procedures were explained to the subjects’ parents, and written informed consent was obtained. The Central Committee on Research Involving Human Subjects (CCMO; file number NL28210.000.09), The Hague, Netherlands, and the Medical Ethics Committees of the UMCUtrecht approved the study protocol.

### Study design

Before the start of the study, the randomization sequence was generated using randomization software (PASW Statistics version 18.0, SPSS Inc, Chicago, IL) by the principal investigator. Participants were randomized over the NP and HP diets with equal amount of participants in both groups. Randomization concealment was guaranteed by using sequentially numbered closed envelopes that contained notes with the letters NP or HP. A copy of the randomization list was kept in a locked safe in the pediatric cardiology department to be opened should an adverse event occur. Patients were randomized in order of recruitment as soon as consent had been obtained. Per patient the closed envelope, together with the patients’ body weight, was send to the department of clinical nutrition on day 1 of the study protocol for preparation of the study diet according to protocol.

At enrolment, a medical history was obtained and physical examination performed. Preoperatively, the cardiopulmonary bypass circuit was primed with standardized, body weight-related amounts of packed cells and albumin, but not steroids. In the OR, all patients received a multiluminal central venous catheter with the tip in the superior or inferior caval vein, a catheter in a peripheral artery for blood sampling and invasive monitoring of the blood pressure, and a nasogastric feeding tube, according to standard cardiac surgical procedures. Additionally, for study purpose, a nasopharyngeal tube was inserted for aspiration of breath samples. General anesthesia was induced using sevoflurane and fentanyl, sporadically combined with midazolam. During anesthesia and surgery, unexpected events, vascular clamping time and total CPB time were noted. After surgery, the patients were transferred to the PICU for postoperative care and ventilated for 4–6 h under mild sedation using propofol (standard dose: 1–2 mg · kg^−1^ · h^−1^) and morphine (standard dose: 10 μg · kg^−1^ · h^−1^). After cessation of sedation they were extubated at the attending anesthetist’s and pediatric intensivist’s clinical judgment. During the entire postoperative intensive care period, medical treatment was instigated by the medical staff of the PICU. Notes were made of the amounts of prescribed sedative and analgesic medication.

**Fig. 1:**
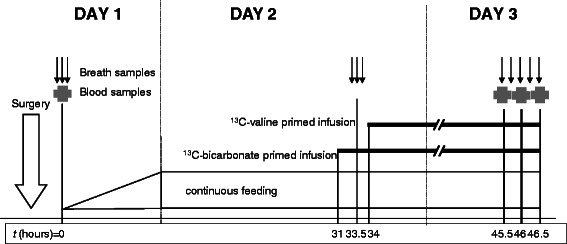
Experimental design. Timing of surgery (open arrow), and sampling of blood (cross) and breath (small arrows). Duration of primed infusions of isotopes is represented by straight lines

After approximately 4 h in the PICU, at time zero (*t* = 0 h), the liquid study diet was administered in increasing amounts via a nasogastric tube with the use of a feeding pump (Kangaroo 324, Kendall Healthcare products, Mansfield, MA) such that the required feeding rate was reached before *t* = 12 h (Fig. 1). The researchers, the parents of the children, and the medical and nursing staff of the PICU were blinded for the composition of the study diets. There were no visible differences between the feeding containers with NP or HP study-diet. The diets consisted of a mixture of whey protein/carbohydrate powder (Hydrolyzed Whey Protein Powder/Maltodextrin Mix), carbohydrate powder (Fantomalt), and fat emulsion (Calogen; all products of Nutricia, Zoetermeer, The Netherlands) dissolved in water. The caloric need was estimated by age-related Schofield equation. Via continuous drip-feeding the protein intake was set at 5 g · kg^−1^ · d^−1^ in the intervention group (high protein, HP) and 2 g · kg^−1^ · d^−1^ in the control group (normal protein, NP). Patients in both groups received a glucose intake of 6 mg · kg^−1^ · min^−1^, with the remaining non-protein calories supplied by fat. Since all patients were fluid restricted after cardiac surgery, the volume of water in which the macronutrients were dissolved was limited at 60 mL · kg^−1^ · d^−1^.

Also, at *t* = 0 h, both a blood and breath sample were taken for measurement of background isotopic enrichment of α-ketoisovalerate (KIV) and ^13^CO_2_, respectively. The end-tidal breath samples for enrichment of ^13^CO_2_ were slowly aspirated through a nasopharyngeal tube with a syringe, and collected in 10 mL sterile, air evacuated, glass tubes with silicon-coated stopper (Tyco Healthcare/Covidien Ltd., Dublin, Ireland). Plasma concentrations of insulin, cortisol, cortisol binding globulin (CBG), and blood urea nitrogen (BUN) were measured.

At *t* = 31 h, NaH^13^CO_3_ (Cambridge Isotope Laboratories Inc, Andover, MA) was continuously infused at a rate of 0.14 μmol · kg^−1^ · min^−1^, after a prime bolus of 12 μmol · kg^−1^, to determine CO_2_ production rate. At *t* = 33.5-34 h, after one blood and three duplicate breath samples were taken for determination of background isotopic enrichments, infusion of [1–13] C-valine (Cambridge Isotopes) was administered at a rate of 0.184 μmol · kg^−1^ · min^−1^ (prime 9.1 μmol · kg^−1^). The isotopes were dissolved in normal saline and infused by volumetric infusion pumps (Infusomat Space Infusion Pump; B.Braun Melsungen AG, Melsungen, Germany), after passage through a 0.20-μm Millipore filter (Minisart; Sartorius AG, Göttingen, Germany). All infusates were tested for pyrogenicity, purity and concentration.

From *t* = 45.5 to 46.5 h, after reaching presumed isotopic equilibrium, 5 concurrent breath samples were collected in duplicate at 15-min intervals. In the same period, at 30-min intervals, three blood samples were collected for measurement of isotopic enrichment of KIV and ^13^C-valine incorporation in albumin. In this 30-min sampling period an extra blood sample was taken for second measurement of plasma concentrations of insulin, cortisol, CBG, and BUN. Also, at *t* = 46 h, as part of the routine clinical assessment of discomfort, the FLACC (face, legs, activity, cry, consolability) score was noted [13].

All blood samples were immediately centrifuged at 4000 rpm for 10 min at room temperature (Labofuge 300; Heraeus Instruments GmbH, Hanau, Germany). After separation, plasma was stored at −20 °C until analysis. At *t* = 46 h, serum glucose concentration was measured bedside (i-STAT r1 analyzer MN300 series; Abbott Laboratories, Chicago, IL). At *t* = 46.5 h, the study was ended, and all children resumed to the standard hospital age-related diets.

### Assays and isotopic analysis

Plasma insulin concentrations were ascertained by using a chemiluminescent immunometry assay on an Immulite analyzer (DPC, Los Angeles, CA) with an intra-assay CV of < 6 %, an inter-assay CV of < 6 %, and a detection limit 15 pmol · L^−1^. Cortisol was measured by chemiluminescent immunoassay on an Immulite analyzer with an intra-assay CV of < 8 %, an inter-assay CV of < 7 %, and a detection limit of 50 nmol · L^−1^. CBG was measured by radio-immuno assay (BioSource Europe S.A., Nivelles, Belgium) with intra-assay CV of 4–5 %, an inter-assay CV of 9–12 %, and a detection limit of 10 mg · L^−1^.

Plasma albumin and BUN concentrations were measured by spectrophotometry (both Roche Diagnostics, Basel, Switzerland).

A detailed description of KIV-measurement was previously described [14]. ^13^C isotopic enrichment in the breath samples was analyzed by an infrared isotope analysis technique (Helifan, Analytic Fischer Instruments, Leipzig, Germany). The ^13^C enrichment was expressed as the atom percentage excess (APE) above baseline.

### Albumin isolation and enrichment measurements

To isolate pure albumin from plasma, we used anti-human serum albumin affinity resin kits (Vivascience-Sartorius Group, Hannover, Germany). Enclosed spin columns were filled with 400 μL affinity resin and 50 μL of thawed plasma. According to the included protocol, the column was washed three times with a tris-buffer and albumin was thereafter eluted from the affinity resin with 0.1 mol · L^−1^ phosphoric acid (acidified to pH 2.5 with HCl). Eluted albumin was precipitated with 700 μL of 2 mol HClO_4_ · L^−1^. A washing step was performed with 0.2 mol HClO_4_ · L^−1^ by re-suspending and precipitating the pellet again. The protein pellet was then hydrolyzed in 140 μL 6 mol HCl · L^−1^ for 22 h at 110 °C. Amino acids (AA) were isolated with the use of a cation-exchange column and then derivatized with ethylchloroformate, and enrichment was measured on a gas chromatograph combustion isotope ratio mass spectrometer (GC/C/IRMS; Delta XP; Thermo Electron, Bremen, Germany) as previously described [15].

### Calculations

The rate of appearance of valine in plasma (Ra_val_), carbon dioxide production (*V*CO_2_ in mmol · h^−1^), and valine oxidation (Oxid_val_) were calculated according to standard equations [16, 17]:

1. Ra_val_ = I_val_ x (E_inf_/E_val_ – 1)
2. *V*CO_2_ = I_bicarb_ x 0.81/E^13^CO_2 bicarbonate plateau_
3. Oxid_val_ = *V*CO_2_ x (E^13^CO_2 valine plateau_ - E^13^CO_2 bicarbonate plateau_)/E^13^C_KIV_

where I_val_ is the infusion rate of labelled valine, E_inf_ tracer enrichment of the labelled valine solution (=99 %), E_val_ valine enrichment in plasma during plateau phase, I_bicarb_ is the infusion rate of labelled bicarbonate, E^13^CO_2_ enrichment of carbon dioxide in the breath samples, and E_KIV_ enrichment in plasma of KIV.

In this model of single amino acid tracer kinetics non-oxidative disposal of valine (NOD_val_) represents protein synthesis (S), and endogenous rate of appearance of valine (Endo-Ra_val_) represents protein breakdown (B) proportionally [16].

(4) S = NOD_val_ (μmol · kg^−1^ · min^−1^) = Ra_val_ – Oxid_val_
(5) B = Endo-Ra_val_ (μmol · kg^−1^ · min^−1^) = Ra_val_ – val(diet)
(6) Protein balance (g · kg^−1^ · d^−1^) = [(4) – (5)]) · 1440/[450 (μmol · g^−1^protein)]

where 1440 is the amount of minutes in 24 h, and 450 μmol · g^−1^ protein represents the estimated contribution of valine to whole body protein [18].

Using plasma KIV enrichment at plateau level, as albumin precursor, we also calculated albumin fractional synthesis rate (FSR_alb_) as described by Verbruggen *et al.* [19]. The FSR_alb_ reflects the fraction of the intravascular albumin pool that is renewed per unit of time (% · d^−1^) and can be calculated by using the following equation:

(7) FSR_alb_ = (E_val-alb_/E_KIV_)/Δ*t* x 100 %

where E_val-alb_ is the enrichment in mole percent excess (MPE) of incorporated valine in albumin, and E_α-kiv_ is the mean enrichment in MPE of the precursor, *i.e.*, plasma KIV. Δ*t* is the time between start of the valine infusion and time of blood sample.

### Statistical analysis and study power

In young children following cardiac surgery, there are no available estimates of mean valine balance with standard deviation, obtained from the same study design and dietary intervention as in our present study. Alternatively, in a previous study in young children following cardiac surgery who were enterally fed with a low protein/normal carbohydrate diet (0.3 g · kg^−1^ · d^−1^ and 7.5 mg · kg^−1^ · min^−1^, respectively), we found net whole-body valine balance of −0.65 (standard deviation: 0.56) μmol · kg^−1^ · d^−1^ on the third day after the operation [20]. Correction of a post-operative negative balance to values > 0 μmol · kg^−1^ · d^−1^ is generally accepted as clinically relevant. Also in the comparison of the effects of protein intakes of 2 and 5 g · kg^−1^ · d^−1^, we consider a statistically significant increase of valine balance by −0.65 μmol · kg^−1^ · d^−1^ to be clinically relevant. Assuming that the previously found SD within groups would also be true in the present study, we calculated that 12 patients per randomization group were needed for sufficient power to detect differences between groups (with α = 0.05, β = 0.8 and power 0.2).

Because the small sample size in the present study was insufficient to assess the assumption of normality, data are presented as median and range. The Mann–Whitney *U* test was used to investigate differences between group medians. P-values less than 0.05 were considered statistically significant. Additionally, based on these results, the Hodges-Lehmann procedure was used to create a confidence interval with a point estimate of median differences between groups. All analyses were performed by using SPSS for WINDOWS statistical software (version 21.0; SPSS Inc, Chicago, IL).

## Results

### Patients

**Fig. 2:**
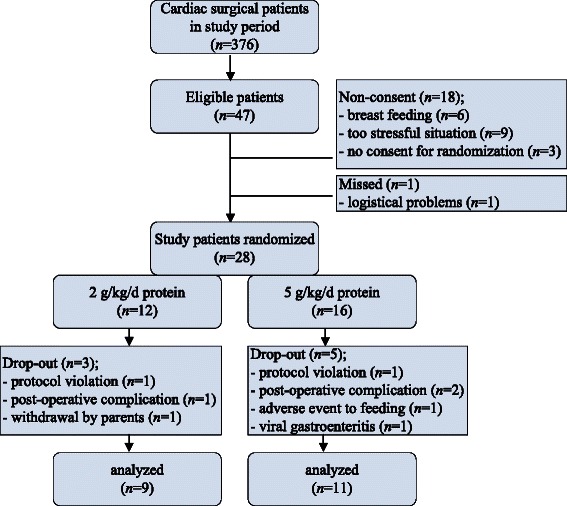
Flow chart eligible patients

We were able to enroll 28 patients in the study who fulfilled the inclusion criteria (Fig. 2). The inclusion rate was 60 % (28/47) of eligible patients. Parents declined consent for reasons of continuation of breast feeding, stressful situation around surgery, and reluctance for randomization of nutrition. Additionally, due to the fact that three patients of the NP group and five of the HP group dropped out of the study, the final analysis of the results comprises nine patients in the NP group and 11 in the HP group. As listed in Table 1, there were no statistically significant differences in baseline characteristics of the NP and HP groups. In the NP group there were 3 patients with Z-score weight-for-height (WFH) < −2.0, compared to 5 patients in the HP group. The range within each group was of comparable width. As a result, the median Z-score of WFH in the HP group was more negative than in the NP group. No differences in aortic clamping time, time on CPB circuit, and priming of the CPB circuit were observed between groups (Table 2). In the postoperative phase, according to randomization, the actual protein and carbohydrate intakes were reached as targeted, with no difference in total caloric intake between groups. Intakes of non-protein calories and fat differed by study design, the latter to achieve isoenergetic diets (Table 3). The contribution to fat intake of the propofol infusion (propofol dissolved in a 10 % fat emulsion at rate 0.1-0.2 mL · kg^−1^ · h^−1^) in the immediate postoperative phase until extubation within 6 h after surgery, was < 0.1 g · kg^−1^ · d^−1^ fat.

**Table 1:** Baseline characteristics of groups

	NP	HP
	(*n* = 9)	(*n* = 11)
Boys/girls	3/6	5/6
Age (mo)	12 (3 to 15)	7 (3 to 14)
Body weight (kg)	7.5 (4.4 to 12.0)	7.1 (3.6 to 9.1)
Body weight (SDS)	−2.2 (−4.4 to 0.8)	−2.1 (−4.1 to 0)
Height (SDS)	−1.3 (−3.2 to 2.1)	−1.0 (−1.9 to 1.3)
Weight for height (SDS)	−1.0 (−2.8 to 0.1)	−1.7 (−2.9 to 0.6)
Diagnosis:		
VSD	5	4
ASD I	2	2
VSD + ASD II	1	3
pAVSD	1	2
Preop O_2_ saturation (%)	99 (94–100)	98 (96–100)

Values are presented as number of subjects or median (range)

There were no statistically significant differences between groups (Mann–Whitney *U* test)

*NP* normal protein (2 g · kg^−1^ · d^−1^) diet, *HP* high protein (5 g · kg^−1^ · d^−1^) diet, *SDS* standard deviation score, *VSD* ventricular septal defect, *ASD I* atrial septal defect type 1, *ASD II* atrial septal defect type 2, *pAVSD* partial atrioventricular septal defect

**Table 2:** Intraoperative aortic clamping time, priming and time on cardiopulmonary bypass circuit

	NP	HP	*P*-value difference between medians^a^	Median difference between groups (95 % CI)^b^
	(*n* = 9)	(*n* = 11)		
Aortic clamping time (min)	35 (20–55)	30 (18–46)	0.71	−2 (−14 to 8)
Time on CPB (min)	59 (36–75)	53 (37–83)	0.37	−7 (−17 to 8)
Priming CPB circuit:				
Erythrocytes (mL · kg^−1^)	140 (100–260)	130 (80–180)	0.18	−25 (−75 to 10)
Plasma (mL · kg^−1^)	0 (0–25)	0 (0–0)	0.41	0 (0 to 0)
Albumin 20 % (mL · kg^−1^)	50 (50–100)	50 (50–75)	0.77	0 (0 to 0)

Values are presented as median (range)

*NP* normal protein (2 g · kg^−1^ · d^−1^) diet, *HP* high protein (5 g · kg^−1^ · d^−1^) diet, *CPB* cardiopulmonary bypass

^a^Mann–Whitney *U* test; ^b^Hodges-Lehmann procedure

**Table 3:** Actual dietary intake during isotopic study

	NP	HP	*P*-value difference between medians^a^	Median difference between groups (95 % CI)^b^
	(*n* = 9)	(*n* = 11)		
Caloric intake (kcal · kg^−1^ · d^−1^)	85 (79–92)	84 (70–87)	0.13	−3 (−8 to 1)
Non-protein calories (kcal · kg^−1^ · d^−1^)	77 (72–84)	65 (53–68)	<0.05	−14 (−19 to −10)
Protein intake (g · kg^−1^ · d^−1^)	2.0 (1.8 - 2.1)	4.7 (4.3 - 5.0)	<0.05	2.8 (2.6 to 2.9)
Protein intake (En%)	9.3 (8.7 - 10.0)	22.8 (21.9 - 24.5)	<0.05	13.6 (13.2 to 14.1)
Carbohydrate intake (mg · kg^−1^ · min^−1^)	5.9 (5.5 - 6.3)	5.7 (5.2 - 6.0)	0.08	−0.2 (−0.5 to 0)
Fat intake (g · kg^−1^ · d^−1^)	4.8 (4.3 - 5.3)	3.5 (2.3 - 3.9)	<0.05	−1.4 (−1.8 to −1.0)

Values are presented as median (range)

*NP* normal protein (2 g · kg^−1^ · d^−1^) diet, *HP* high protein (5 g · kg^−1^ · d^−1^) diet, *En%* energy percent

^a^Mann–Whitney *U* test; ^b^Hodges-Lehmann procedure

The study protocol was violated one time in each group (premature removal of sample line and accidental removal of nasogastric feeding tube, respectively), and in three patients the study was stopped due to postoperative complications (sternum dislocation, cardiac tamponade, and junctional ectopic tachycardia). In the HP group, two patients had gastric residues and/or diarrhea, which in one patient was caused by viral gastroenteritis. One patient in the NP was withdrawn from the study by the parents due to personal circumstances.

At *t* = 45.5 to 46.5 h, data regarding valine kinetics and endocrine response could be obtained and analyzed in nine patients in the NP group and 11 patients in the HP group, respectively. In either group, from one patient oxidation data are missing, due to sampling error of breath samples.

### Protein metabolism

There was, by study design with higher dietary protein intake and consequent higher valine intake in the HP group compared to the NP group, a significant difference in total valine flux (turnover) between groups. We observed no statistically significant differences in non-oxidative disposal of valine, representing protein synthesis, endogenous rate of appearance of valine, representing protein breakdown, and net whole-body protein balance between groups (Table 4). Additionally, the fractional synthesis rate of albumin and plasma albumin concentrations did not show differences between groups. In the HP group, compared to the NP group, there was statistically significant higher valine oxidation with higher BUN concentrations [difference between groups (95 % CI): 0.94 (0.90 to 1.88) μmol · kg^−1^ · min^−1^, p < 0.05, and 3.3 (2.2 to 4.9) mmol · L^−1^, *p* < 0.05, in the HP and NP group, respectively].

**Table 4:** Absolute and relative contribution to total valine flux of endogenous Ra of valine (total flux corrected for exogenous supply), valine oxidation, non-oxidative disposal of valine, total valine and body protein balance, fractional synthesis rate of albumin, serum albumin, and blood urea nitrogen

	NP [% flux]	HP [% flux]	*P*-value difference between medians^a^	Median difference between groups (95 % CI)^b^
	(*n* = 9)	(*n* = 11)		
Total valine flux (μmol · kg^−1^ · min^−1^)	2.82 (2.48 to 3.37) [100]	4.26 (3.25 to 5.85) [100]	<0.05	1.38 (1.01 to 1.90)
Valine dietary intake (μmol · kg^−1^ · min^−1^)	0.91 (0.86 to 0.97) [32.4]	2.20 (2.01 to 2.30) [52.0]	<0.05	1.28 (1.22 to 1.34)
Endogenous valine Ra (μmol · kg^−1^ · min^−1^)	1.90 (1.63 to 2.48) [67.7]	2.06 (1.12 to 3.64) [48.1]	0.50	0.15 (−0.23 to 0.61)
Valine oxidation (μmol · kg^−1^ · min^−1^) ^c^	0.68 (0.29 to 1.07) [22.1]	1.58 (0.38 to 2.93) [35.2]	<0.05	0.94 (0.90 to 1.88)
NOD of valine (μmol · kg^−1^ · min^−1^) ^c^	2.26 (1.85 to 2.73) [77.9]	2.73 (0.94 to 3.36) [64.9]	0.28	0.37 (−0.55 to 0.88)
Valine balance (μmol · kg^−1^ · min^−1^)	0.24 (−0.20 to 0.63)	0.54 (−0.73 to 1.75)	0.57	0.29 (−0.67 to 1.17)
Protein balance (g · kg^−1^ · d^−1^)	0.78 (−0.65 to 2.00)	1.73 (−2.35 to 5.59)	0.57	0.94 (−2.15 to 3.74)
FSR albumin (% · d^−1^)	8.0 (7.1 to 12.0)	9.3 (6.1 to 13.9)	0.20	1.3 (−0.7 to 3.0)
Albumin (g · dL^−1^)	3.9 (3.7 to 4.5)	3.7 (3.4 to 4.2)	0.13	−0.2 (−0.4 to 0.1)
BUN (mmol · L^−1^)	4.0 (2.7 to 5.6)	7.1 (4.4 to 11.7)	<0.05	3.3 (2.2 to 4.9)

Values are presented as median (range) absolute [and relative] contribution to total valine flux with estimated differences between groups (confidence interval)

*NP* normal protein (2 g · kg^−1^ · d^−1^) diet, *HP* high protein (5 g · kg^−1^ · d^−1^) diet, *CI* confidence interval, *NOD* non-oxidative disposal, *Ra* rate of appearance, *FSR* fractional synthesis rate, *BUN* blood urea nitrogen

^a^Mann–Whitney *U* test; ^b^Hodges-Lehmann procedure; ^c^based on *n* = 8 and *n* = 10 in the NP and HP group, respectively (see text)

### Endocrine response

**Fig. 3:**
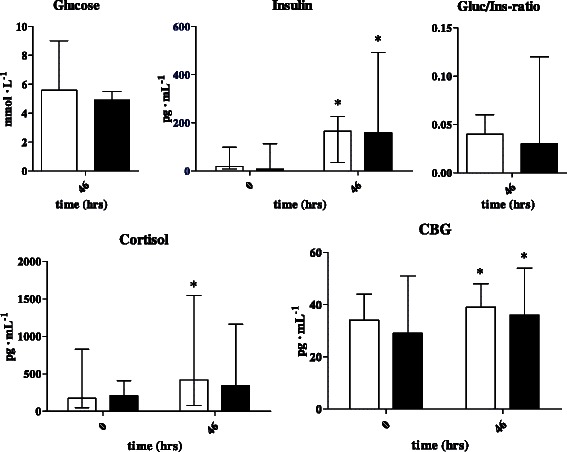
Low-high bar graphs (median with range) of glucose, insulin (including gluc/ins-ratios), cortisol, and CBG concentrations at *t* = 0 and *t* = 46 for NP group (*n* = 9, white bars) and HP group (*n* = 11, grey bars). NP, normal protein (2 g · kg^−1^ · d^−1^) diet; HP, high protein (5 g · kg^−1^ · d^−1^) diet; Gluc, glucose; Ins, insulin; CBG, cortisol binding globulin. MDD, mean difference between groups of delta within groups between time points with 95 %-confidence interval (Hodges-Lehman procedure) and *p*-value (Mann–Whitney *U* test)

Between the NP and HP group, we found no statistically significant differences in plasma concentrations of glucose at *t* = 46 h, and plasma concentrations of insulin, cortisol and CBG at *t* = 0 h and *t* = 46 h (Fig. 3). Also, there were no differences between groups of the delta plasma concentration between time points of these parameters. There were no statistically significant differences between cortisol-to-CBG ratios between groups.

### Clinical observations

In both groups, the median FLACC score at *t* = 46 h was 0 with range 0 to 7. There was no statistically significant difference between median body temperatures [37.5 °C (range: 37.0-38.1) in the HP group, compared to 37.3 °C (36.8-37.8) in the NP group; difference between groups with 95 % CI: 0.2 °C (−0.2 to 0.5), *p* = 0.22].

## Discussion

It is not clear what the optimal protein intake should be in infants and young children following cardiac surgery with the use of CPB. In the present study, we studied the effect on whole-body protein synthesis (WBPS) and balance of a high-protein diet (HP, 5 g · kg^−1^ · d^−1^), compared to an isocaloric normal-protein diet (NP, 2 g · kg^−1^ · d^−1^) in this patient population. However, the observed differences between groups with regard to these primary outcome parameters showed such wide confidence intervals without statistical significance, that we could not exclude the occurrence of a predefined clinically relevant difference in valine balance of >0.65 μmol · kg^−1^ · d^−1^ in either direction. Therefore, we conclude that our study was underpowered, due to high dropout rates in both groups together with a large variation within the groups, with infants being at positive and negative protein balance. Three patients in the NP group and five in the HP group did not complete the isotopic measurements. Although in only two cases in the HP group the premature termination of the study was related to gastrointestinal adverse effects (one of which was caused by a viral gastroenteritis), we cannot rule out potential bias in the reported results associated with the use of a completers’ analysis.

In this article we present our observations as new hypothesis generating data.

We observed positive valine balance in both study groups [0.54 (−0.73 to 1.75) and 0.24 (−0.20 to 0.63) μmol · kg^−1^ · min^−1^ in the HP and NP group, respectively, *p* = 0.57], with valine synthesis rate of 2.73 (range: 0.94 to 3.36) and 2.26 (1.85 to 2.73) μmol · kg^−1^ · min^−1^ in the HP and NP diet, respectively (*p* = 0.28). These observations are in agreement with a systematic review of protein balance studies in mixed PICU’s, that shows that in critically ill children a positive protein balance can be achieved by moderate intake of calories 57 kCal · kg^−1^ · d^−1^ and protein 1.5 g · kg^−1^ · d^−1^ [10]. In a small study in neonates following general surgery, Duffy *et al.* observed an improvement of the protein balance due to decreased endogenous protein breakdown at higher protein intake of 3.9 ± 0.5 g · kg^−1^ · d^−1^, compared to normal protein intake of 2.3 ± 0.4 g · kg^−1^ · d^−1^ [21]. However, in that study, the high protein intake group also received a higher amount of total calories compared to the normal protein group (91 and 75 kCal · kg^−1^ · d^−1^, respectively), and therefore the observed decrease in proteolysis and effect on net protein balance may not be attributable solely to higher protein intake. In a recent study, Vlaardingerbroek *et al.* showed that in very low birth weight infants, an increase of protein intake from 2.4 g · kg^−1^ · d^−1^ to 3.6 g · kg^−1^ · d^−1^ resulted in higher protein synthesis rates, but also in increased amino acid oxidation and urea production [22]. In a systematic review in low birth weight infants, Premji *et al.* identified 5 studies that showed improved nitrogen accretion rates with high intakes of 4–6 g · kg^−1^ · d^−1^ balanced protein, compared to age-related reference protein intake of 3 g · kg^−1^ · d^−1^ [23]. However, those studies also found adverse metabolic effects such as azotemia and metabolic acidosis [23]. Despite the fact that five of the included studies predating 1995, in a time in which the amino acid balance of feeding solutions did not meet present-day standards, we cannot rule out the possibility that metabolic acidosis has occurred in our patients, since we did not routinely measure blood pH as part of the study. Finally, in a study in burned children, Patterson et al. reported a non-significant increase in muscle and skin protein synthesis together with a linear and significant increase of urea production in patients with high (2.92 ± 0.19 g · kg^−1^ · d^−1^), compared to normal (1.15 ± 0.28 g · kg^−1^ · d^−1^) protein intake [24].

Also in our study, in the HP diet compared to the NP diet, we observed significantly higher valine oxidation rate [difference between groups with 95 %-CI: 0.94 (0.90 to 1.88) μmol · kg^−1^ · min^−1^, *p* < 0.05] and plasma urea levels [3.3 (2.2 to 4.9) mmol · L^−1^, *p* < 0.05]. We consider the observed higher oxidation rate and concomitant ureagenesis plausible explanations of the metabolic fate of surplus amino acids. These findings strengthen the hypothesis that in this population protein synthesis is already maximally stimulated by a protein intake of 1.5-2 g · kg^−1^ · d^−1^, and that excess dietary protein is oxidized.

In our study, there were no differences of FSR_alb_ between the two groups. The observed values in our study were lower than those in a study in infants following general surgery (16 ± 2.2 % · d^−1^) [19] or in malnourished children with infection (19.8 ± 2.2 % · d^−1^) [25]. It is known that in critically ill children, lower albumin synthesis rates may be observed as the result of increased synthesis rates of acute phase proteins such as CRP and fibrinogen, at the expense of decreased synthesis of selected proteins (*e.g.* albumin, transferrin, prealbumin, retinol-binding protein, and fibronectin) [26]. Moreover, in our study in children following cardiac surgery, the CPB circuit was primed with albumin (median [range]: 1.5 [1.0 to 4.2] g · kg^−1^) as part of standard procedure, resulting in relatively high plasma albumin concentrations in the NP and HP groups, which might further have suppressed albumin synthesis in our patients.

In our study, we have infused the bicarbonate and valine isotopes for 15 and 12 h, respectively (Fig. 1). As a consequence of this long infusion period, we might have induced recycling of the labelled valine, resulting in the release of the isotopic tracer from previously synthesized proteins into the blood compartment. This might have resulted in higher plasma ^13^C-valine enrichment, with subsequent underestimation of total valine flux, endogenous Ra_val_ and NOD_val_. We estimated that this might have affected results in both groups proportionally.

## Conclusions

In conclusion: in spite of underpowering of the study with regard to short-term effects on protein synthesis and balance of a high protein diet (HP, 5 g · kg^−1^ · d^−1^), compared to a normal protein diet (NP, 2 g · kg^−1^ · d^−1^) in young children following cardiac surgery, our data support the hypothesis that in this population the metabolic fate of surplus protein in the HP diet, compared to the NP diet, was oxidation and ureagenesis. We cannot rule out a possible detrimental effect of metabolic acidosis due to high protein intake in the HP group, since we did not routinely measure blood pH. Therefore, a high protein diet (5 g · kg^−1^ · d^−1^) is not a meaningful, yet even potentially hazardous strategy in young children following cardiac surgery.

## Abbreviations

AA: Amino acid
CHD: Congenital heart disease
CPB: Cardiopulmonary bypass
FSR: Fractional synthesis rate
HP: High protein
KIV: α-ketoisovalerate
LOD: Limit of detection
NOD: Non-oxidative disposal
NP: Normal protein
PICU: Pediatric intensive care unit
Ra: Rate of appearance
